# A deep learning framework for identifying Alzheimer's disease using fMRI-based brain network

**DOI:** 10.3389/fnins.2023.1177424

**Published:** 2023-08-08

**Authors:** Ruofan Wang, Qiguang He, Chunxiao Han, Haodong Wang, Lianshuan Shi, Yanqiu Che

**Affiliations:** ^1^School of Information Technology Engineering, Tianjin University of Technology and Education, Tianjin, China; ^2^Tianjin Key Laboratory of Information Sensing and Intelligent Control, School of Automation and Electrical Engineering, Tianjin University of Technology and Education, Tianjin, China

**Keywords:** fMRI, Alzheimer's disease, 2D-CNN, phase synchronization index, ROI

## Abstract

**Background:**

The convolutional neural network (CNN) is a mainstream deep learning (DL) algorithm, and it has gained great fame in solving problems from clinical examination and diagnosis, such as Alzheimer's disease (AD). AD is a degenerative disease difficult to clinical diagnosis due to its unclear underlying pathological mechanism. Previous studies have primarily focused on investigating structural abnormalities in the brain's functional networks related to the AD or proposing different deep learning approaches for AD classification.

**Objective:**

The aim of this study is to leverage the advantages of combining brain topological features extracted from functional network exploration and deep features extracted by the CNN. We establish a novel fMRI-based classification framework that utilizes Resting-state functional magnetic resonance imaging (rs-fMRI) with the phase synchronization index (PSI) and 2D-CNN to detect abnormal brain functional connectivity in AD.

**Methods:**

First, PSI was applied to construct the brain network by region of interest (ROI) signals obtained from data preprocessing stage, and eight topological features were extracted. Subsequently, the 2D-CNN was applied to the PSI matrix to explore the local and global patterns of the network connectivity by extracting eight deep features from the 2D-CNN convolutional layer.

**Results:**

Finally, classification analysis was carried out on the combined PSI and 2D-CNN methods to recognize AD by using support vector machine (SVM) with 5-fold cross-validation strategy. It was found that the classification accuracy of combined method achieved 98.869%.

**Conclusion:**

These findings show that our framework can adaptively combine the best brain network features to explore network synchronization, functional connections, and characterize brain functional abnormalities, which could effectively detect AD anomalies by the extracted features that may provide new insights into exploring the underlying pathogenesis of AD.

## 1. Introduction

Alzheimer's disease (AD), the most common cause of dementia, is a progressive, degenerative brain disease (Prince, 2018) which is the main cause of disability among older adults, affecting around 50 million people worldwide (Prince et al., 2019). By 2050, it is estimated that 1 in every 85 individuals will be affected by this condition, and the number of affected individuals is projected to double within the next 20 years (Sarraf and Tofighi, 2016). Alzheimer's disease (AD) has been associated with several common abnormalities in the patient's brain: (1) amyloid-related imaging abnormalities (ARIA) (Salloway et al., 2022); (2) damaged areas of nerve fibers and the occurrence of neurofibrillary tangles (Moloney et al., 2021); (3) disconnection of brain network connectivity compared to normal aging individuals (Rosenberg et al., 2020). These pathological or physiological heterogeneities directly or indirectly affect the diagnosis of AD.

To overcome these challenges, the diagnosis of Alzheimer's disease requires meticulous medical evaluation, patient history records, mental state examinations (MMSE), as well as various neurobiological and physical examinations (Duc et al., 2020). Additionally, functional magnetic resonance imaging (fMRI) has rapidly advanced due to its safety, non-invasiveness, and high spatial resolution, making it the most commonly used method for analyzing regular brain changes, different activities, and studying functional connectivity and synchronization between brain regions (Ibrahim et al., 2021). Currently, the identification of AD primarily relies on resting-state functional magnetic resonance imaging (rs-fMRI), which differs from task-based functional magnetic resonance imaging (task-based fMRI). Resting-state fMRI does not affect the individual's ability to recognize and execute task instructions, making it highly useful for studying the cognitive decline caused by AD. During these tests, patients remain in a resting state where they neither engage in any activities nor have any specific thoughts, making the data acquisition task simple and enabling the assessment of routine brain changes (Tong et al., 2017). The rs-fMRI network has been demonstrated to be highly sensitive to AD (Zhang et al., 2021). Magnetic resonance imaging (MRI) and functional magnetic resonance imaging (fMRI) techniques reveal lower MRI signal intensity, reduced brain tissue and cortical volume, enlarged ventricles, affecting brain regions and neural networks associated with cognition, memory, planning, and decision-making (Janghel and Rathore, 2021), ultimately leading to cognitive decline symptoms and decreased complexity of brain networks in AD patients (Cai et al., 2018). Therefore, distinguishing the visual differences between AD data and images from elderly participants with traditional aging effects requires in-depth information and knowledge, combined with additional clinical clues for accurate data classification (e.g., MMSE) (Duc et al., 2020). However, throughout the process, a tool or algorithm is needed to classify imaging data based on rs-fMRI, such as analyzing rs-fMRI data to establish brain network features for quantitative analysis, and differentiate healthy individuals from AD patients for appropriate treatment approaches.

Synchrony has been widely used on the brain network analysis, including the correlation coefficient, Granger causality, phase synchronization index (PSI), et al. (Dauwels et al., 2010b; Cai et al., 2018). Particularly, PSI, as a nonlinear synchrony analysis, can quantify the relationship between the instantaneous phases of AD signals (Zheng and Zhang, 2013; Szymanski et al., 2017). Cai et al. (2018) constructed PSI network by EEG signals, and found that the brain network connectivity of AD patients was abnormal and the small-world property was weakened. Nobukawa et al. (2020) estimated the functional connectivity of AD brains by building brain networks using the phase lag index through EEG signals, and found that brain connectivity and complexity reduced in AD patients. According to the previous synchrony analysis, functional networks of multiple signals could be constructed (Chen et al., 2019b) based on complex network and graph theory. Functional connectivity can describe the relationship between multiple channels in spatially remote brain regions, and quantify their synchronous relationship in the complex brain system (Bi et al., 2020; Sporns, 2022). Therefore, it has been widely applied to fMRI to understand the brain network structure, development and evolution of AD. Zhang et al. (2019) established brain network from fMRI data by using Pearson correlation, and found the loss of small-world properties and the destruction of whole brain tissue network in AD brain. Si et al. (2019) proposed a mutual information brain network model, and revealed that AD brain had characteristics of a longer average time to process information transfer between brain regions and a reduction in data processing capacity than healthy control (HC). Note that functional networks can represent information transmission between different brain regions.

As one of the mainstream deep learning algorithms, convolutional neural networks (CNNs) have made tremendous progress in various fields, such as image recognition and clinical diagnosis (Chen et al., 2019a). CNNs can learn features automatically from large-scale datasets, and then identify global and local patterns (Samek et al., 2016; Mahmud et al., 2018). Li et al. (2019) proposed a hybrid convolutional and recurrent neural network for more detailed hippocampus analysis using structural MRI to recognize AD brain and obtained a high accuracy rate. Venugopalan et al. (2021) used stacked de-noising auto-encoders to extract features from clinical and genetic data of AD, and used CNN for imaging data, then found that hippocampus, amygdala brain areas and the rey auditory verbal learning test (RAVLT) were significant changes in AD brain. Despite variable structures of CNNs, the deep features extracted from CNNs were used to explain their strong recognition power (Bi et al., 2020; Gao et al., 2022). One aim of this study was to find possible explanations of the learned features based on the connectivity matrices derived from PSI matrix of fMRI signals.

In this paper, a new framework, which combined the PSI, CNN and support vector machine (SVM) measures, is proposed and used for recognition and detection of AD from fMRI. Topological and deep features are extracted to quantify the different local and global patterns between AD and HC brain networks. The rest of this paper are organized as follows: In Section 2, the ADNI dataset and data preprocessing are described. In Section 3, the proposed methods for AD classification are illustrated, including methods for constructing brain networks using PSI, CNN structure, deep features and topological feature extraction. Section 4 presents the analytical results, which consists of deep and topological features analysis, statistical analysis, classification analysis, followed by a discussion in Section 5. The conclusions are drawn in Section 6.

## 2. Materials

### 2.1. Subjects

In this study, data used in the preparation of this article were obtained from the Alzheimer's Disease Neuroimaging Initiative (ADNI) database (adni.loni.usc.edu/) (Jack Jr et al., 2008). The primary objective of ADNI is to assess whether a combination of sequential magnetic resonance imaging (MRI), positron emission tomography (PET), other biomarkers, as well as clinical and neuropsychological evaluations, can be used to measure the progression of mild cognitive impairment (MCI) and early-stage Alzheimer's disease (AD). To date, over 1,000 scientific publications have used ADNI data. A number of other initiatives related to AD and other diseases have been designed and implemented using ADNI as a model. Additionally, the fMRI data were collected by a total 3T fMRI data of 118 patients with AD and 127 healthy older adults (HC) were downloaded from the Alzheimer Disease Neuroimaging Initiative (Jack Jr et al., 2008). The main characteristics of the subjects are reported in Table 1, which presents the baseline clinical and demographic variables of both groups. Additionally, the fMRI data were collected by 3T Philips fMRI scanner, where the specific scanning parameters are as follows: TR/TE is 3000ms/30ms, flip Angle is 80°, imaging matrix is 64 × 64, voxel size is 3.31mm × 3.31mm × 3.31mm, 48 slices.All original image files are available to the general scientific community. In this table, AD represents Alzheimer's disease patients, NC represents normal controls, MMSE refers to Mini-Mental State Examination, and CDR represents Clinical Dementia Rating.

**Table 1:** Demographics and neuropsychologica.

	**Age (mean ±SD)**	**Female/ male**	**MMSE (mean ±SD)**	**CDR**
AD (*n* = 118)	72.7 ± 7.01	56/62	21.0 ± 3.52	1
NC (*n* = 127)	74.6 ± 7.44	63/64	29.0 ± 1.15	0

### 2.2. fMRI preprocessing

**Figure 1:**
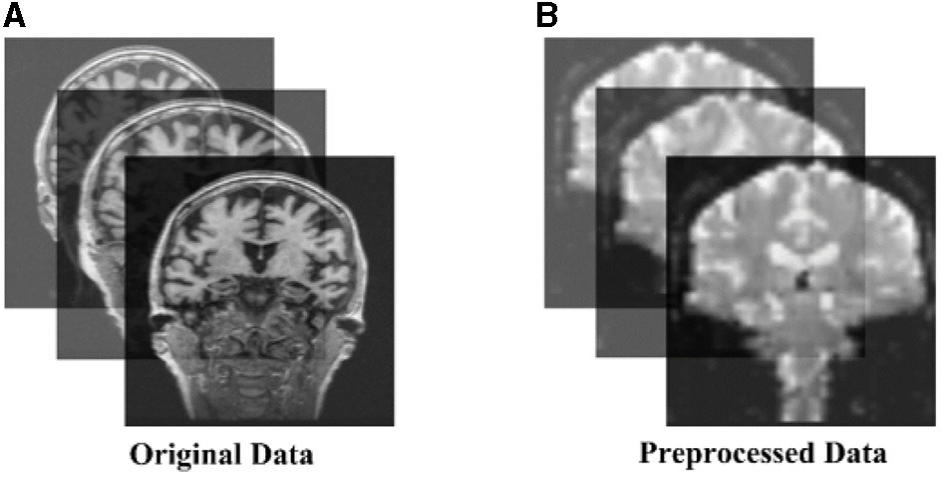
The **(A)** original and **(B)** pre-processed fMRI.

All preprocessing was performed using the Matlab toolbox SPM12 (https://www.fil.ion.ucl.ac.uk/spm/software/spm12/) and CONN (https://www.nitrc.org/search/?type_of_search=group&q=conn). (1) Data discard: The first 10 time points were discarded for scanner calibration and for subjects to get used to the circumstance; (2) Slice timing: The time offset between adjacent slices were removed by the time slice correction image; (3) Realignment: Motion artifacts generated on different images between individual subjects were eliminated; (4) Normalization: The images after realignment were spatial normalized to the standard EPI template with 3 × 3 × 3 resolution; (5) Smoothing: The normalized images were further spatially smoothed with a Gaussian kernel of 6mm full width at half maximum (FWHM); (6) Filtering: The temporal filtering (0.01Hz<f<0.08 Hz) was applied to the time series of each voxel to reduce the effect of low-frequency drifts and high-frequency noise such as respiratory and cardiac rhythms; (7) ROI signal extraction: the original image was converted into ROI signals using the AAL atlas based 116 Brain Regions (AAL-116) template. Figure 1 shows the raw and pre-processed fMRI.

## 3. Methods

### 3.1. Functional brain networks construction

For the identification of functional and structural changes in the brain of AD patients through constructing brain networks, the brain network, as a graph, has two crucial components: nodes and edges. Therefore, this study employs the phase synchronization method to investigate the phase coupling of signals within and between regions of interest (ROIs), which are extracted using the AAL atlas, serving as the nodes. The functional connectivity between two signals is calculated using the phase synchronization index (PSI) and represents the edges for constructing the brain functional network. Due to the sensitivity of Phase Synchronization Index (PSI) to low-frequency signals, which reflect the regulation and synchronization of neural activity, utilizing phase synchronization analysis to process low-frequency signals in fMRI aligns well with relevant concepts and mechanisms in neurophysiology. Therefore, it is a commonly used metric for measuring the strength of functional connectivity in networks. PSI describes the instantaneous phase relationship between time series. Through the Hilbert transformation of the signal sequence (Thuraisingham et al., 2012), that is, the one-dimensional real signal is transformed into a two-dimensional signal on the complex plane. *H*_*x*_(*t*) represents Hilbert transform from *x*(*t*), which can be defined as follows (Yu et al., 2020):


(1) $$H_{x}(t)=\frac{1}{\pi }pv\int_{-x}^{\infty }\frac{x(\tau )}{t-\tau }d\tau$$

where *pv* is the Cauchy principal value. The instantaneous amplitude *A*(*t*) and the instantaneous phase φ(*t*) can be computed by:


(2) $$\begin{array}{l} A\left(t\right)=\sqrt{{\left[x\left(t\right)\right]}^{2}+{\left[H_{x}\left(t\right)\right]}^{2}} \end{array}$$

(3) $$\begin{array}{l} \varphi \left(t\right)=\arctan\frac{H_{x}\left(t\right)}{x\left(t\right)} \end{array}$$

**Figure 2:**
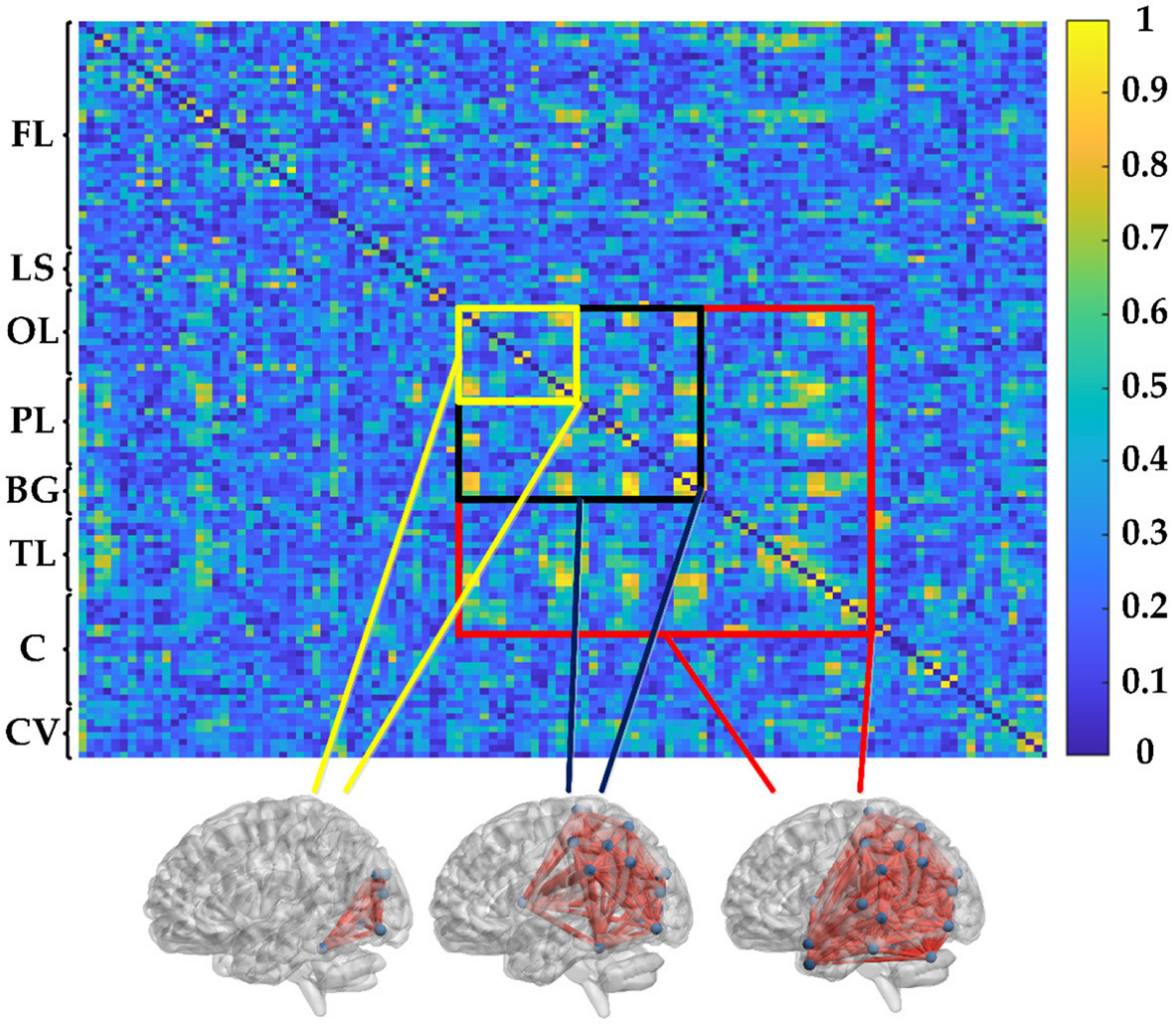
PSI matrix of regional brain network: FL: frontal lobe, LS: limbic system, OL: occipital lobe, PL: parietal lobe, BG: basal ganglia, TL: temporal lobe, C: cerebellum, and CV: cerebellar vermis.

The phase difference between two signals is defined as:


(4) $$\begin{array}{l} \text{Δ}\varphi \left(f_{m},f_{n},t\right)=m\varphi \left(f_{m},t\right)-n\varphi \left(f_{n,t}\right) \end{array}$$

where *f*_*m*_ and *f*_*n*_ are the center frequencies of two signals. Besides, *m* and *n* are integers that should satisfy the condition *m*·*f*_*n*_ = *n*·*f*_*m*_. The phase difference is calculated by setting *m* = *n* = 1. Therefore, the PSI can be defined as:


(5) $$PSI(f_{m},f_{n})=\left|\langle e^{j-\left(\Delta \varphi \left(f_{m},f_{n},t\right)\right)}\rangle \right|$$

where 〈·〉 refers to the averaging across time. Obviously, PSI is a symmetrical measure (i.e. *PSI*_*XY*_ = *PSI*_*YX*_) and within the range [0, 1]. According to graph theory, the PSI matrix in this study can be transformed into an unweighted binary adjacency matrix by applying a given threshold (= 0.3). In the representation of the functional network graph, only connections with PSI values greater than the threshold are realized, resulting in corresponding entries of the matrix being 1, while others are 0. Therefore, the functional connectivity network can be established by PSI, as shown in Figure 2. The 116 × 116 connection matrix is displayed through BrainNet Viewer toolbox (http://www.nitrc.org/projects/bnv/) from full views, according to three brain regions of different sizes of connectivity.

### 3.2. Convolutional neural network

Convolutional neural network (CNN) is a deep learning network, which can maintain the original data hierarchical structure and apply translation invariance to convert information to feature information for classification (Azulay and Weiss, 2018). It is designed to learn the latent and intrinsic features from images in a supervised way. These features are appropriate in categorizing the anatomical structures and diagnosing the abnormal structures. CNN has a hierarchical structure, where each layer learns feature representations at different levels of abstraction. The lower-level convolutional layers primarily learn low-level features such as edges and textures, while deeper convolutional layers gradually learn higher-level features such as shapes and structural components. This hierarchical feature learning ability enables CNN to effectively capture semantic information in image data. In this study, the convolutional operation in CNN is used to generate extractable deep features, thereby analyzing the object features and texture features of the input data from an image perspective. It provides insights into the input data from both global and local perspectivesIn the convolution layer of a CNN, there are many neurons connected spatially and share the weight and bias, and it uses a set of convolutional kernels to obtain the feature mapping of the input image (Wang et al., 2021). In the convolution layer, the input image is mapped with a set of kernels to generate a new feature map *C*_*k*_ and this process is called convolution. The feature value at location (*x, y*) in the *k*^*th*^ feature map of *i*^*th*^ layer, *w*_*k*_ is the weight of the kernel, *b*_*k*_ is bias of the kernel, $C_{k}^{i}\left[x,y\right]$ is calculated:


(6) $$\begin{array}{l} C_{k}^{i}\left[x,y\right]=w_{k}^{i}I^{i}\left[x,y\right]+b_{k}^{i} \end{array}$$

where *I*^*i*^[*x, y*] is the value at location (*x, y*) of input image to the *i*^*th*^ layer.

Pooling layers are used to reduce the dimensionality of computed feature maps Singh et al. (2020). In CNN models, three commonly used pooling operations are max pooling, min pooling, and average pooling (Satti et al., 2020; Tasnim et al., 2021). After a series of convolutional and pooling operations, the output of the final convolutional layer is propagated to the classification layer. Let *R*^*l*^ be the output of the last convolutional layer, and *R*^*l*^ is computed as follows:


(7) $$\begin{array}{l} R^{l}=f\left(I^{l}\right)=f_{relu}\left(\sum_{k}w_{k}*f\left(I^{l-1}\right)\right) \end{array}$$

where *k* represents the number of kernels used in the last convolutional layer, *w*_*k*_ denotes the weights of the kernels, *f*(*I*^*l*−1^) represents the activation values of the (*l* − 1) convolutional layer, and *f*_*relu*_ represents the computation through the ReLU activation function.

Finally, the classification layer uses the softmax activation function to predict the class of an input image. The softmax function calculates the class probability value (Wang et al., 2018):


(8) $$\begin{array}{l} f_{soft\max}\left(R^{l}\right)=\frac{e^{R^{l}}}{\sum e^{R^{l}}} \end{array}$$

**Figure 3:**
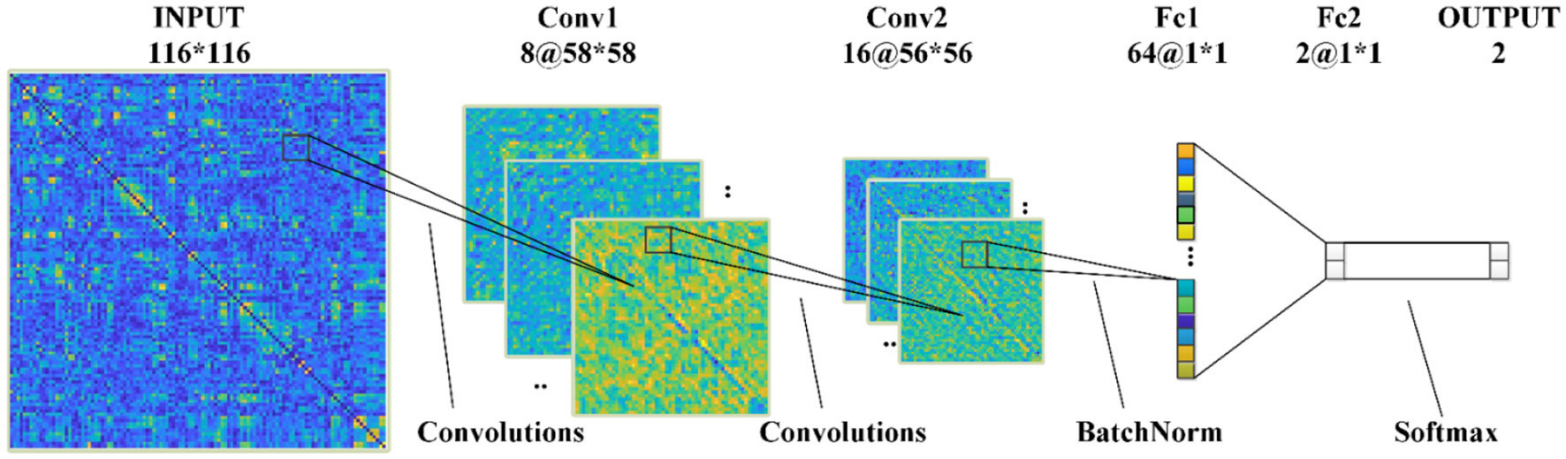
The 2D-CNN model proposed in this paper.

Figure 3 shows the structures of proposed CNNs in this paper, which has two convolutional (Conv) layers and two fully connected (Fc) layers: (1) In the first Conv layer, there are 8 Conv kernels/filters with a kernel size of 3 × 3, stride of 2, pad of 2, and max pooling size of 2; (2) In Conv2, there are 16 Conv kernels/filters with a kernel size 3 × 3, stride 1 and pad 1; (3) ReLU activation functions are used for all Conv/Fc layers and sigmoid activation functions for output layers, respectively.

### 3.3. Feature extraction

Deep features can extract both local and global feature information from images, while brain network topological features provide connectivity patterns and functional associations between brain regions. Combining these two types of features can better describe the functional and structural information in brain imaging data, thereby enhancing the performance of disease classification. Additionally, integrating deep features with brain network features can provide a more comprehensive and intuitive visual analysis, aiding researchers in understanding the intrinsic characteristics and brain functionality of brain imaging data. This section describes the eight topological features to be extracted: degree (DG), node betweenness (NB), edge betweenness (EB), assortativity (AS), clustering coefficient (CC), global efficiency (GE), local efficiency (LE) and average characteristic path length (APL), see details in Supplement file. In addition, eight deep features F1-F8 were automatically extracted from the last convolutional layer of 2D-CNN.

### 3.4. Statistical analysis

Single-factor analysis of variance (ANOVA) was used to assess the statistical differences between the AD and HC groups. ANOVA returns several statistical measures, including the sum of squares (SS), degrees of freedom (df), mean squares (MS = SS/df), *F*-value, and *P*-value. The *F*-value is the ratio of the between-group mean squares (MSB) to the within-group mean squares (MSW), indicating the extent of between-group differences (Wang et al., 2015). The *P*-value is inversely related to the *F*-value and represents the probability of error when between-group differences are not significant. In statistics, it is generally considered significant when P<0.01. A larger *F*-value or a smaller *P*-value indicates more significant between-group differences, and vice versa.

In addition, the receiver operating characteristic (ROC) curve was used to visually evaluate the ability of these features to distinguish AD patients from normal controls. Through variance analysis, the between-group differences were found to be significant. This statistical method summarizes the performance of two classifiers over a range of possible thresholds from 0 to 1. Sensitivity refers to the true positive rate, while specificity refers to the true negative rate.


(9) $$\begin{array}{l} Sensitivity=\frac{TP}{TP+FN} \end{array}$$

(10) $$\begin{array}{l} Specificity=\frac{TN}{TN+FP} \end{array}$$

Among them, false negatives (FN) are the number of AD patients misclassified as normal controls, and false positives (FP) are the number of normal controls misclassified as patients. True positives (TP) and true negatives (TN) are the counts of correctly identified AD patients and normal controls, respectively. Accuracy quantifies the total number of correctly classified subjects and is defined as follows:


(11) $$\begin{array}{l} Accuracy=\frac{TP+TN}{TP+FP+TN+FN} \end{array}$$

The area under the ROC curve (AUC) represents the performance of the classification. For a perfect classifier, the AUC is 1, while an AUC of 0.5 indicates a test with no value (Ferraris, 2019).

### 3.5. Application

The application process for AD detection is as follows:

(1) Preprocessing of fMRI Images: Apply standard preprocessing steps such as motion correction, slice-timing correction, and spatial normalization to the fMRI images. These steps enhance the data quality and provide the raw data for subsequent analysis.
(2) Extraction of ROI Signals using AAL Atlas: Extract the average time series signals from each region of interest (ROI) using the Automated Anatomical Labeling (AAL) atlas. These signals represent the brain activity in the corresponding regions.
(3) Calculation of PSI to Measure Functional Connectivity: Calculate the PSI to quantify the functional connectivity between brain regions. Construct a connectivity matrix based on PSI values and extract 8 topological features (DG, NB, EB, AS, CC, GE, LE, FL) from the network. These measures represent the overall connectivity and information exchange capabilities of the brain network.
(4) Analysis and Extraction of Deep Features using CNN: Train a CNN model on the PSI dataset to learn discriminative patterns in fMRI images. Extract 8 deep features from the learned CNN model. These features provide intuitive representations of object characteristics and texture features within the PSI network, offering new insights into the affected regions in the brain associated with AD from a global and local perspective.
(5) Feature Selection and Classification: Select relevant features from the topological features derived from the PSI brain network and the deep features extracted from the CNN model for classification. Evaluate the impact of using the same features or different feature combinations on the classification accuracy. Employ a SVM classifier to classify AD and non-AD samples. Apply 5-fold cross-validation to ensure the robustness of the classification results. The training and test sets are split with a ratio of 4:1. This process aids in model training and evaluation while ensuring the reliability of the classification results.

**Figure 4:**
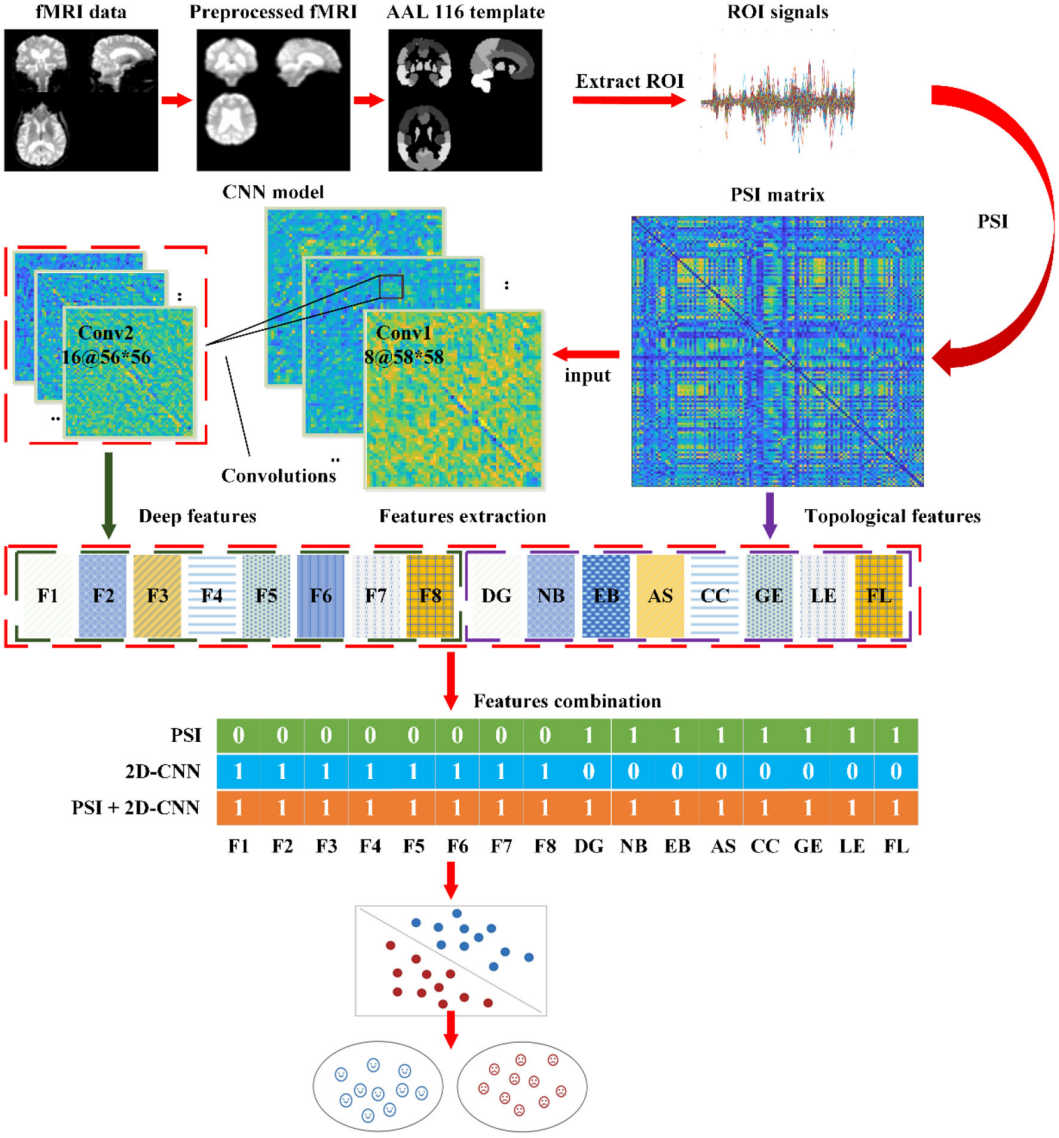
The automatic detection process for AD.

The intelligent detection process is illustrated in Figure 4.

## 4. Results

### 4.1. Analysis of brain network

**Figure 5:**
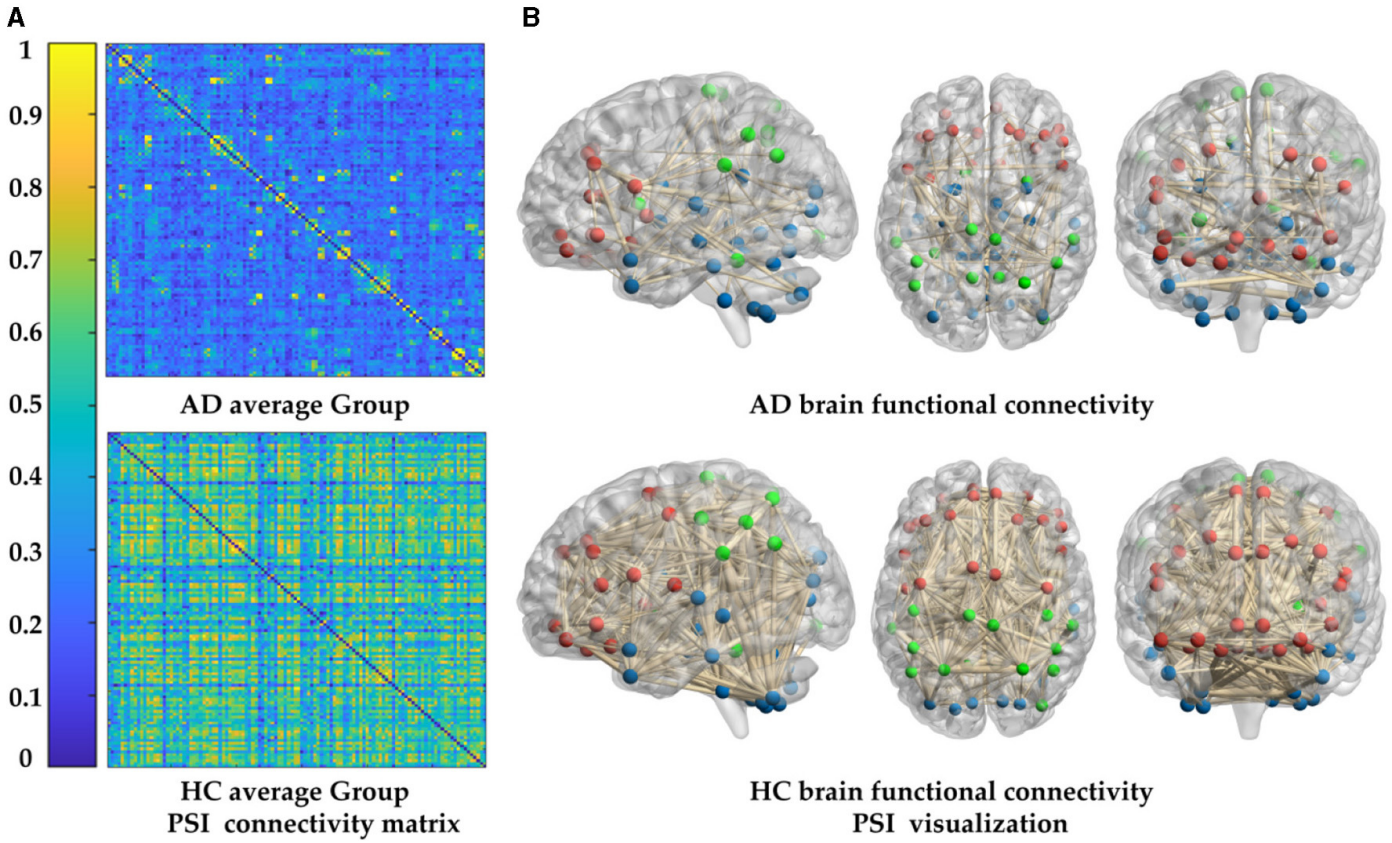
Comparison of whole-brain functional connectivity between the AD and HC group: **(A)** connectivity matrices of the two groups. **(B)** Connectivity full views (frontal, profile, top) of the two groups.

To study the cognitive impairment-related brain mechanisms of AD, the PSI was used to construct the functional connectivity after selecting the same threshold, so the adjacency matrix was obtained (Figure 5A), and the brain network connection corresponding to the matrix was displayed through BrainNet Viewer toolbox from full views (frontal, profile, top), as shown in Figure 5B. Compared to the HC group, the brain networks of the AD group were abnormal. To be specific, the values of PSI matrix of AD group were ranged in [0, 0.4], which were much smaller than that of the HC group ranged in [0.6, 1], reflected in the colder color of the PSI matrix in AD group. Correspondingly, connections between nodes in the AD group were sparser and weaker than that in the HC group, which were shown by the thickness and density of the connecting lines, especially in the frontal lobe (FL), parietal lobe (PL) and basal ganglia (BG), marked by red and green color. That is, the AD group had weaker ability of information transmission and less activation in local brain regions. This partial loss of functional connectivity across brain regions results from degenerative changes in the AD brains.

**Figure 6:**
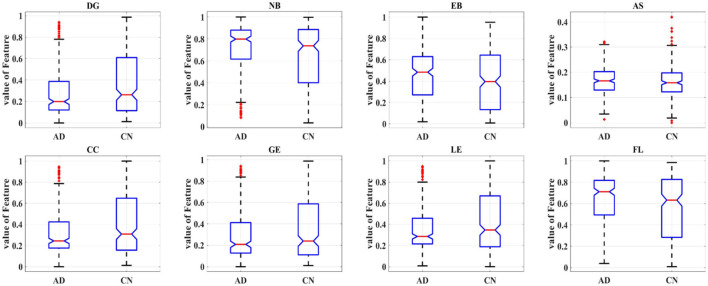
Box plot of one-way ANOVA analysis of the eight topological features.

In order to further study the difference between the AD and HC group, eight topological features of brain network matrix: DG, NB, EB, AS, CC, GE, LE, APL were extracted and performed one-way ANOVA analysis, as shown in Figure 6, Table 2. In Figure 6, box indicates the first and third quartiles, red line indicates the median, whiskers mark the minimum and the maximum, notches indicate the 95% confidence interval, and the red crosses indicate outliers. Moreover, all the values of features in this figure were normalized to the range of [0, 1]. All the topological features except for the AS presented significant differences between AD and HC groups, indicated by the *P*-values of the seven features smaller than 0.01. The values of DE, CC, LE and GE of the AD group are smaller than that of the HC group, suggesting that the connectivity, aggregation and compensatory capacity of the AD network was decreased, and thus the integration ability between AD brain regions was poor; while the values of NB, EB and APL of AD group increased, indicating the small-world property of brain networks was weakened, the information transfer capacity and the rate of information transmission between network nodes of AD network were decreased.

**Table 2:** Results of ANOVA analysis of eight topological features.

**Features**	***F*-values**	***P*-values**	**AD(mean±SD)**	**CN(mean±SD)**
DE	23.97	1.21e-06	0.29 ± 0.24	0.37 ± 0.28
NB	25.52	5.57e-07	0.70 ± 0.23	0.63 ± 0.28
EB	32.93	1.42e-08	0.45 ± 0.23	0.39 ± 0.26
AS	0.137	0.710	0.16 ± 0.05	0.16 ± 0.06
CC	19.31	1.28e-05	0.33 ± 0.23	0.40 ± 0.28
GE	22.42	2.64e-06	0.29 ± 0.24	0.37 ± 0.28
LE	20.58	6.72e-06	0.36 ± 0.22	0.43 ± 0.27
APL	24.90	7.62e-07	0.62 ± 0.25	0.55 ± 0.29

### 4.2. Analysis of 2D-CNN

**Figure 7:**
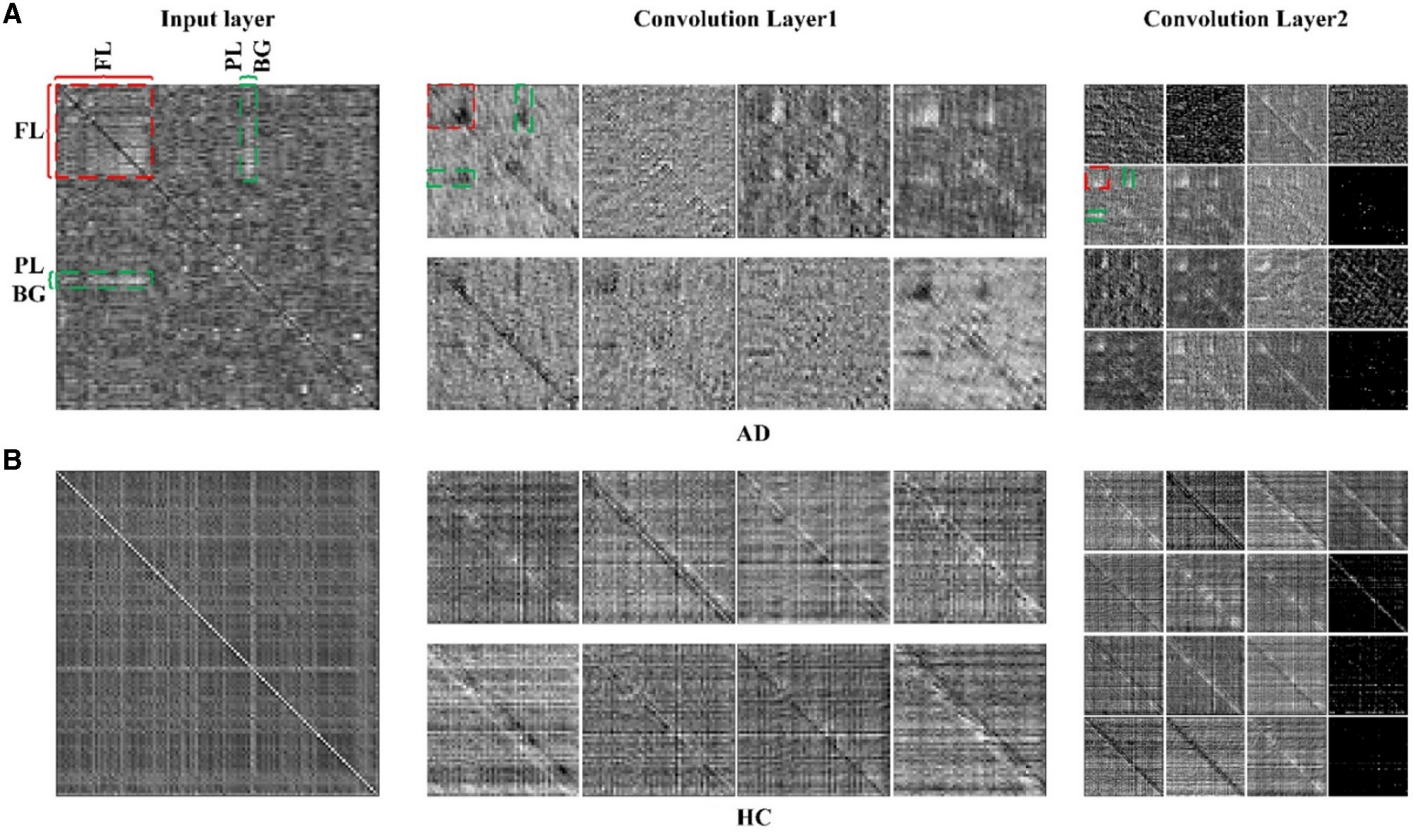
The feature maps of 2D-CNN for **(A)** AD group and **(B)** HC group. Panels on the left, middle and right represented the input layer, convolutional layer 1 and convolutional layer 2 of 2D-CNN model.

To illustrate the effectiveness of CNN on classifying the brain connectivity of AD and HC, and determine the certain lesion region in the AD brain image, the visualization feature maps of convolutional layers by CNN were plotted in Figure 7 to exhibit the abnormality of functional connectivity of AD patients. Figures 7A, B showed the feature maps of AD and HC PSI matrix, respectively. Feature maps generated from convolutional layers 1 and 2 of the CNN model were extracted to show how the convolutional layers work. In each convolutional layer, there were multiple kernels, for which a feature map could be generated by sliding the filters over the PSI matrix (convolution). Therefore, the features map dimension changes drastically from one convolutional layer to the next according to the filters number. In Figure 7, the left, middle and right panels represented the input layer, convolutional layer 1 and convolutional layer 2, respectively. After different convolution operation in convolutional layers, the feature map changed, such as the dimension of features maps of different layers. For example, the input layer was 116 × 116, and convolutional layer 1 and convolutional layer 2 were 58 × 58 and 29 × 29, respectively. However, object features and texture features can preserve certain key information of the original input data of the PSI matrix. Compared to the HC group, the regions of FL, PL, and BG of AD in convolutional layer 1 were much lighter in color, indicating abnormalities in connectivity may be in these brain regions. The same anomaly also occurred in convolutional layers 1 and 2, marked by red and green colors. Thus, the comparison of the feature maps of different layers of the CNN and the exploration of the feature maps could reveal some interesting insights.

**Figure 8:**
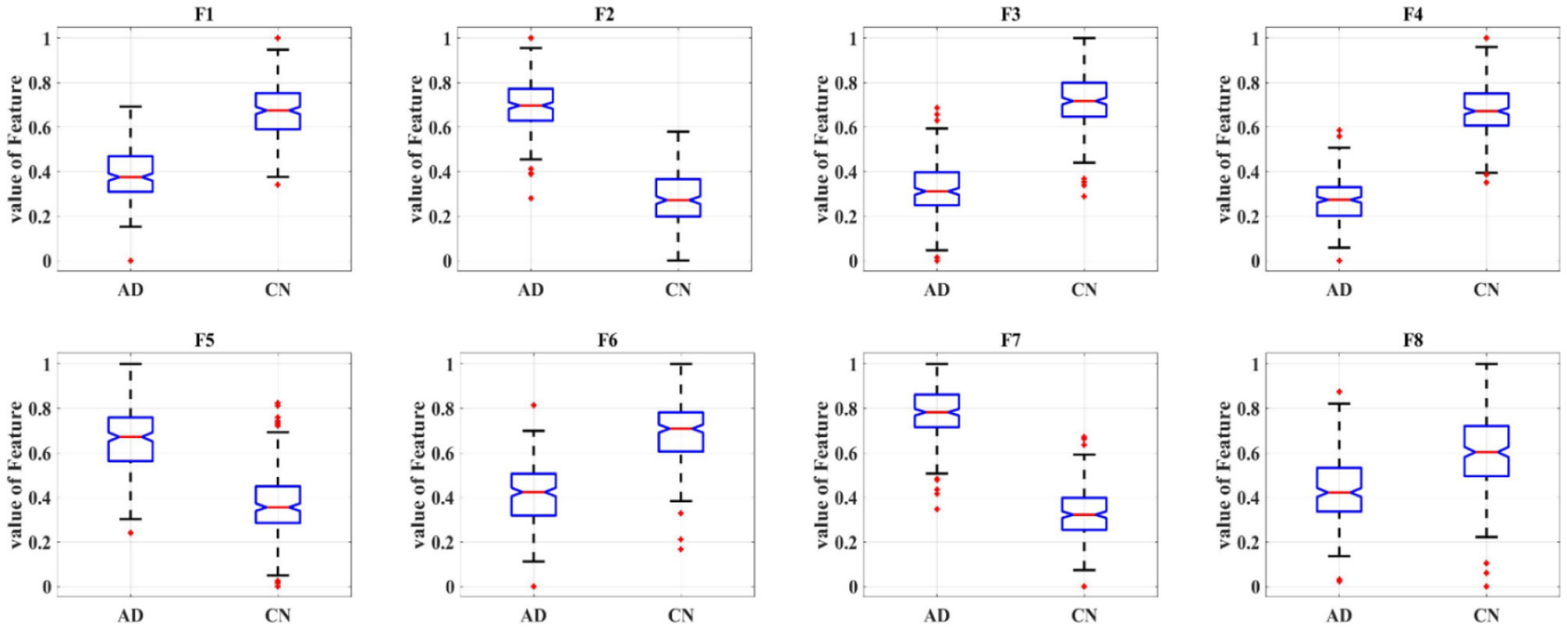
Box plot of one-way ANOVA analysis of the eight deep features.

Eight deep features, which could be divided into two categories: object features and texture features, extracted from the last convolutional layer of the CNN: after normalization, F1-F8 were further extracted and one-way ANOVA analysis was performed to characterize the abnormality of AD brain, as shown in Figure 8, Table 3. All the eight topological features, had obvious group differences with *P*-values smaller than 0.01. Compared to the HC group, F1, F3, F4, F6 and F8 of the AD group were decreased, while F2, F5, F7 of were increased. Notably, the deep features in Table 3 could better display the local and global abnormal regions in AD brain than topological features in Table 2 with larger *F*-values and smaller *P*-values statistically.

**Table 3:** Results of ANOVA analysis of eight deep features.

**Features**	***F*- values**	***P*- values**	**AD (mean ±SD)**	**CN (mean ±SD)**
F1	714.40	4.23e-96	0.39 ± 0.11	0.66 ± 0.14
F2	1600.74	7.84e-153	0.69 ± 0.10	0.27 ± 0.11
F3	1305.12	3.35e-137	0.31 ± 0.11	0.71 ± 0.12
F4	1851.37	2.02e-164	0.27 ± 0.09	0.67 ± 0.11
F5	483.42	4.99e-74	0.66 ± 0.14	0.36 ± 0.15
F6	514.36	2.80e-77	0.41 ± 0.13	0.69 ± 0.13
F7	1872.49	2.43e-165	0.77 ± 0.11	0.32 ± 0.11
F8	130.11	9.47e-27	0.43 ± 0.14	0.59 ± 0.17

### 4.3. Correlation analysis of features

**Figure 9:**
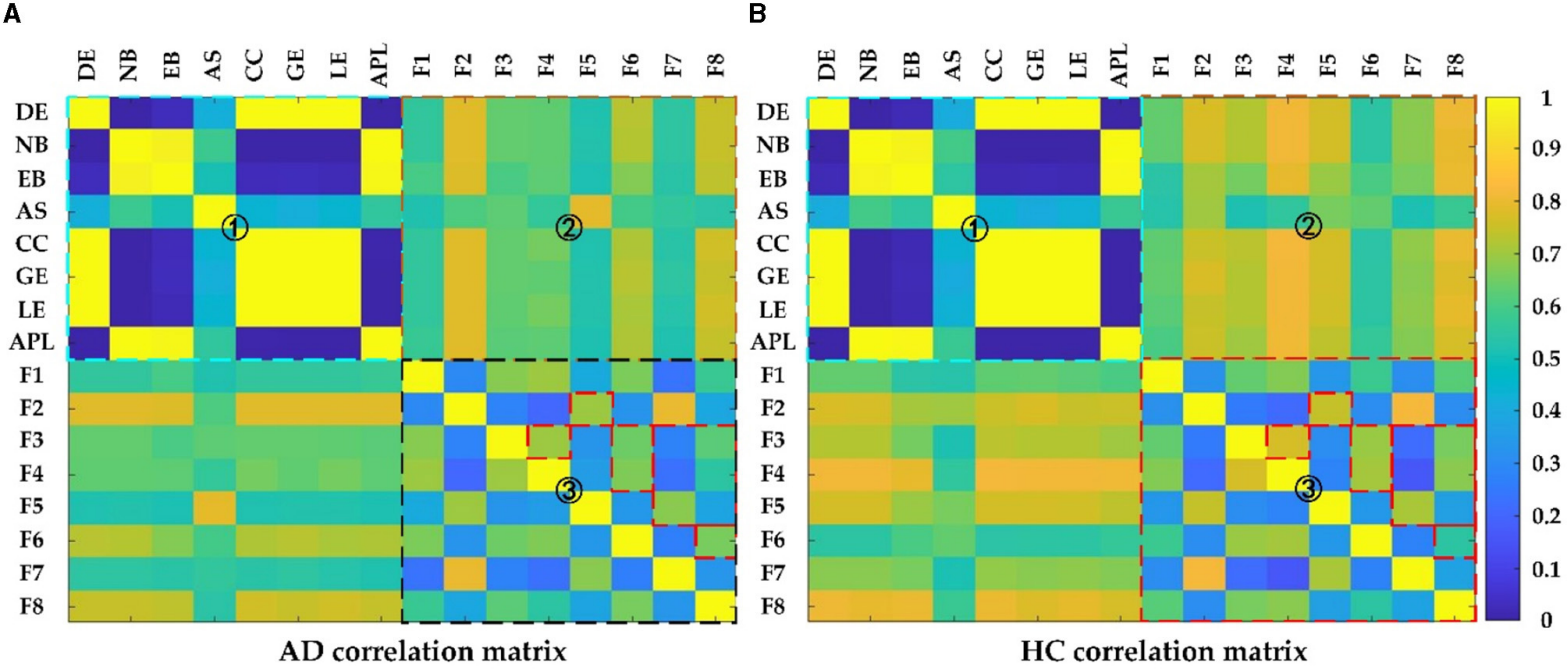
The Pearson correlation matrix of **(A)** AD and **(B)** HC group. Region 1 shows correlation between topological features; Region 2 shows correlation between topological features and deep features; Region 3 shows correlation between deep features.

To gain more insight into the relationship between the different measures, the correlations between those measures were calculated using Pearson correlation. Figure 9 showed the correlation between the deep features and the topological features in AD and HC, where the colder the color, the weaker the correlation, and vice versa. Seen from Figure 9, the correlation matrix could be divided into four parts: top left, bottom right, and top right which was symmetrical with the bottom left part, which represented the correlation between any two topological features: DG, NB, EB, AS, CC, GE, LE, APL, the correlation between any two deep features: F1 F8 and the correlation between topological features and deep features, respectively. For the part of one, it could find that there was strong correlation among the topological features: (1) DE, CC, GE and LE; (2) NB, EB and APL; For the part of three, the correlation between deep features in the HC group was much larger than in theAD group, especially for F2 with F5, F3 with F4/F6/F8, and F5 with F7/ F8; for the part of two, the correlations of deep features F2, F6, F8 with the topological features except AS were relatively strong. Clearly, the reduced feature correlation in AD patients compared to the HC group is due to the effect of reduced inter-regional brain correlations. In addition, the relevance of deep features and topological features can capture the changes in the global pattern of AD from the structural and functional brain network loss and convolutional kernels, and thus can more effectively characterize AD than topological or deep features alone.

**Figure 10:**
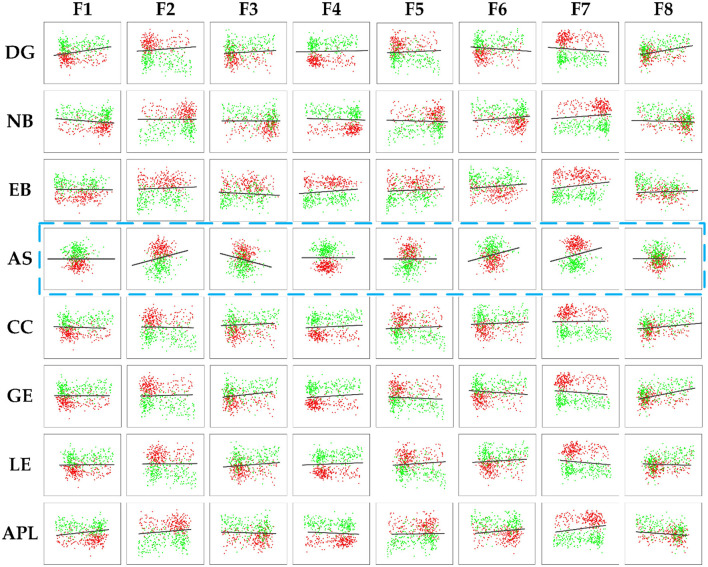
The correlation results between the deep features and the topology features of brain connectivity, where the blue dashed box represents topology feature AS with no significant difference (*P*-values>0.05). The black line denoted the discrimination of different groups.

To further demonstrate the relevance between the deep and topological features and investigate the effect on distinguishing AD from HC, scatter plots are plotted to display the correlation results between the deep and topological features in Figure 10, which was composed of 64 subfigures, where each row represented the eight deep features and each column represented the eight topological features. After normalization, all eight deep features were correlated with any of the eight topological measures, which indirectly verifies the effect of deep features. In each subplot, the red and green points represented the AD and HC group, respectively. In order to find the difference between the two groups of data more intuitively, the black line was fitted to show the discrimination of different groups. When two features were combined, the normalized values of the features were clustered in a distribution between the two groups and can thus be easily discriminated. Interestingly, the topological feature AS, which has no significant group differences originally, could become effective attributing to depth features (marked with blue rectangle), thereby suggesting that the CNN model can learn some features that represent the global patterns of the brain network that are not described by the topological features.

To further quantitatively investigate whether the correlation between the deep and topological features could effectively distinguish AD from HC, the classification accuracy is shown in Tables 4, 5. The corresponding accuracy of each subplot could distinguish between AD and HC, with higher accuracy for the combined features formed by the deep features F1, F2, F3, F4 and F7 with topological features. In summary, when data passing through successive CNN layers, the complexity of the data decreases as the size of the output data decreases, but the correlation with the topological features is preserved, indicating that the global and local patterns on the brain network are preserved during CNN learning. Therefore, it is feasible to improve the classification accuracy of AD and HC by extracting the depth features of the last convolutional layer.

**Table 4:** Results of topology features and the deep features.

	**Sensitivity**	**Specificity**	**AUC**	**ACC**
DE	85.45 ± 1.398	64.19 ± 1.398	72.68 ± 2.210	74.82 ± 2.321
NB	91.39 ± 2.235	75.75 ± 2.235	83.44 ± 2.137	83.57 ± 2.410
EB	95.69 ± 1.001	77.15 ± 1.001	80.48 ± 1.101	86.42 ± 1.012
AS	68.39 ± 0.136	76.23 ± 1.015	65.14 ± 2.014	72.31 ± 2.134
CC	84.89 ± 1.052	57.55 ± 1.052	64.11 ± 2.310	71.22 ± 1.353
GE	88.77 ± 3.020	74.07 ± 3.020	80.67 ± 1.012	81.42 ± 2.111
LE	83.87 ± 2.020	70.07 ± 2.020	65.03 ± 2.307	76.97 ± 2.221
APL	84.62 ± 3.368	86.80 ± 0.368	80.74 ± 3.112	85.71 ± 1.121
F1	97.45 ± 0.018	79.90 ± 1.117	91.10 ± 1.032	88.67 ± 1.109
F2	100.0 ± 0.000	93.83 ± 2.046	91.64 ± 2.125	97.02 ± 1.323
F3	96.52 ± 3.106	88.55 ± 3.607	96.25 ± 2.315	94.04 ± 2.032
F4	98.57 ± 1.095	94.46 ± 1.038	97.34 ± 2.023	97.01 ± 1.119
F5	97.56 ± 1.016	76.01 ± 2.027	87.64 ± 1.448	87.40 ± 1.018
F6	97.65 ± 2.154	72.45 ± 4.131	89.19 ± 2.018	85.47 ± 2.216
F7	99.63 ± 1.083	93.26 ± 1.274	96.74 ± 1.204	96.37 ± 1.112
F8	84.86 ± 2.718	54.58 ± 3.055	73.78 ± 1.566	70.50 ± 2.049

**Table 5:** The results of classification accuracy between the deep features and the topology features.

**Topology features**	**Deep features**							
	**F1**	**F2**	**F3**	**F4**	**F5**	**F6**	**F7**	**F8**
DG	85.69 ± 0.038	94.02 ± 0.025	93.58 ± 0.33 9	4.88 ± 0.011 8	8.77 ± 0.057	79.80 ± 0.047	94.88 ± 0.026	68.16± 0.068
NB	87.18 ± 0.017	96.15 ± 0.028	95.08 ± 0.030	95.72 ± 0.013	86.84 ± 0.053	83.55 ± 0.038	96.16 ± 0.022	68.17 ± 0.063
EB	88.89 ± 0.030	97.22 ± 0.027	94.01 ± 0.031	97.01 ± 0.008	89.05 ± 0.038	84.83 ± 0.014	96.58 ± 0.017	70.52 ± 0.062
AS	89.32 ± 0.027	96.48 ± 0.014	94.44 ± 0.035	96.79 ± 0.015	87.54 ± 0.029	85.90 ± 0.015	96.37 ± 0.021	70.09 ± 0.047
CC	89.21 ± 0.028	97.43 ± 0.016	94.22 ± 0.039	97.02 ± 0.014	87.77 ± 0.028	86.12 ± 0.026	97.31 ± 0.037	69.99 ± 0.052
GE	87.11 ± 0.029	97.22 ± 0.014	93.42 ± 0.032	96.97 ± 0.015	88.80 ± 0.024	86.33 ± 0.024	96.57 ± 0.012	70.42 ± 0.049
LE	89.32 ± 0.071	96.57 ± 0.024	94.44 ± 0.035	95.73 ± 0.016	87.74 ± 0.033	86.47 ± 0.028	98.27 ± 0.022	69.88 ± 0.053
APL	88.35 ± 0.062	97.53 ± 0.017	94.26 ± 0.014	97.12 ± 0.014	89.68 ± 0.027	85.90 ± 0.021	97.42 ± 0.031	69.88 ± 0.046

### 4.4. Classification analysis

**Figure 11:**
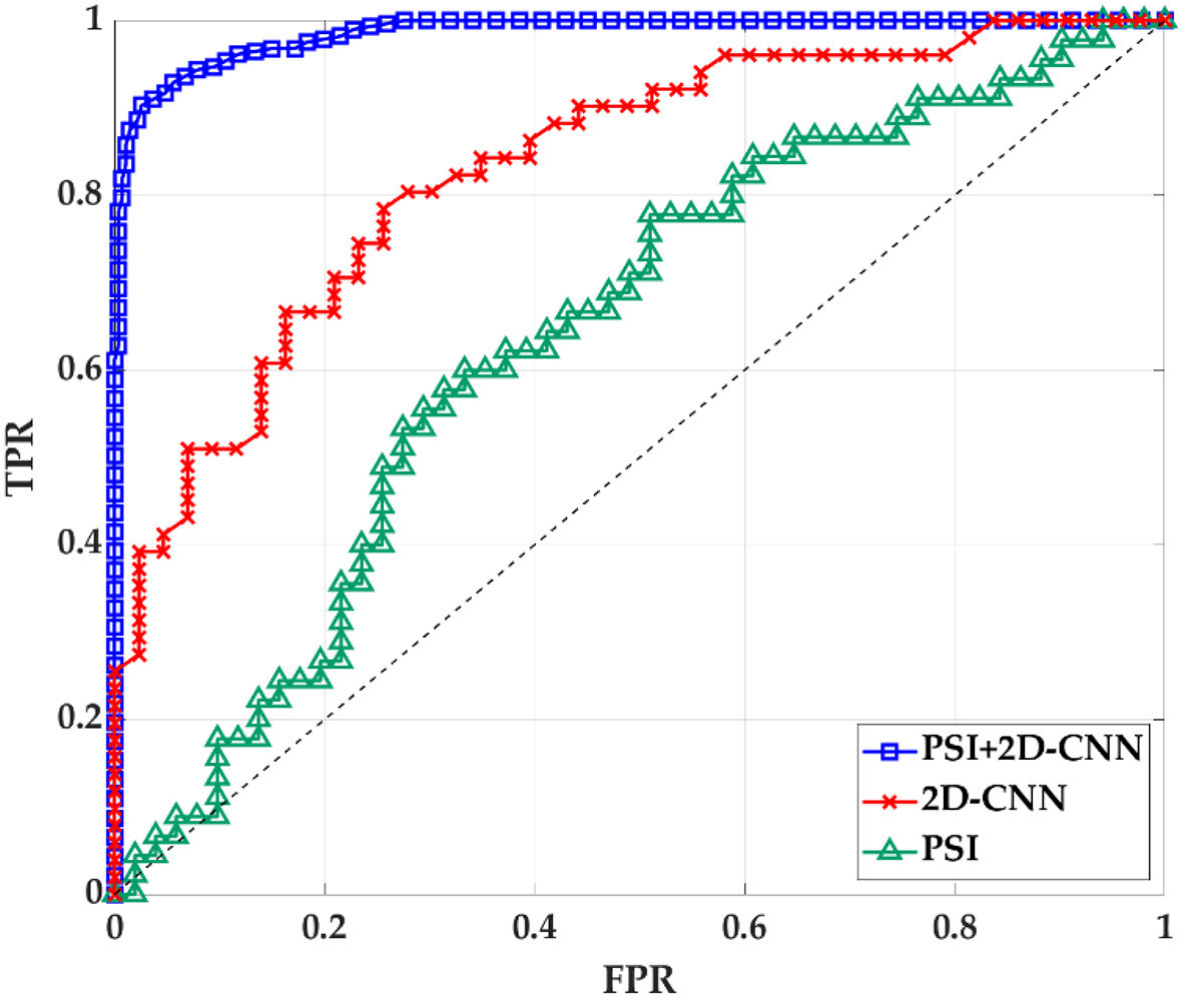
The ROC curve of PSI brain network features, 2D-CNN deep features and PSI and 2D-CNN combination features. TPR is true positive rate. FPR is false positive rate.

The results in the previous section show that there is a certain correlation between TF and BF. Therefore, PSI can be combined with 2D-CNN for classification recognition. After 5-fold crossover, eight topological features and eight depth features are combined with these sixteen features to be fed into an SVM classifier for classification. As shown in Figure 11, Table 6, the three methods of topological features and deep features could distinguish between AD and HC, but the classification by topological features is generally effective. The combination of hybrid method had the best ACC, AUC, SEN and SPE for classifying AD and HC. It indicated that the accuracy of AD recognition can be greatly improved by combining the brain structural features extracted by PSI brain network and the whole brain global pattern features extracted by 2D-CNN. All of them could be used to explore the underlying pathogenesis of AD, which may provide new insights for analysis.

**Table 6:** ACC and AUC classified by SVM after using 8 features for 2D-CNN models and PSI brain network.

**Method**	**SEN (%)**	**SEP (%)**	**AUC (%)**	**ACC (%)**
2D-CNN	96.942 ± 0.132	94.724 ± 0.324	88.859 ± 0.006	95.833 ± 0.340
PSI	71.224 ± 0.023	72.660 ± 0.313	63.965 ± 0.018	71.942 ± 0.100
PSI+2D-CNN	99.124 ± 0.054	98.614 ± 0.335	99.899 ± 0.061	98.869 ± 0.107

## 5. Discussion

Alzheimer's disease is a progressive and incurable neurodegenerative disease, which leads to degradation of cognitive (Chhatwal et al., 2018). Although the pathological mechanisms underlying the neuropathological changes in AD remain unclear, some neuropathological hallmarks have been reported, such as neuritic extracellular amyloid plaques and intracellular neurofibrillary tangles, which would lead to energy slowing down, decrease of complexity and synchrony, and disconnection of the connectivity (Dauwels et al., 2010a). By complex networks theory and graph theory, recent research on brain functional networks extracted from fMRI (Table 7) and the structural networks extracted from MRI have shown that AD patients have different degrees of local or even global topology abnormalities and “disconnection” symptoms (Xu et al., 2008; Sanz-Arigita et al., 2010; Córdova-Palomera et al., 2017; Khatri and Kwon, 2022; Xing et al., 2022).

**Table 7:** Methods for establishing brain networks using fMRI data and conclusions obtained.

**Khatri and Kwon (2022)**	**fMRI SMRI**	**ADNI**	**Pearson correlation analysis**	90*90	**The most affected brain region in AD for all patient classification analysis was mainly located on the middletemporal gyrus, the hippocampus, and the amygdala area followed by other brain regions**.
Xu et al. (2008)	fMRI	Medical College of Wisconsin	Phase shift index	Not mention	The AD group existed asynchrony indicated the more asynchrony exists between spontaneous low frequency components.
Córdova-Palomera et al. (2017)	fMRI	NorCog	Instantaneous phase synchronization	26*26	In the AD group, both static (putamen, dorsal and default-mode)and dynamic (temporal, frontal-superior and default-mode), along with decreased global metastability.
Xing et al. (2022)	fMRI	ADNI	Persistent homology	90*90	The AD group showed a high residence time and a higher window ratio in a weak connection state, which may be because patients with AD have not established a firm connection.
Sanz-Arigita et al. (2010)	fMRI	Alzheimer Center of the VU University Medical Center	Synchronization likelihood	116*116	The regional synchronization reveals increased AD synchronization involving the frontal cortices and generalized decreases located at the parietal and occipital regions.

Additionally, some consistent conclusions of AD networks were obtained that connectivity between brain regions decreased (Si et al., 2019; Zhang et al., 2019) and small-world attribute declined (Yu et al., 2018), specific brain hubs degenerated, clustering coefficients reduced and path lengths closed to the values of random networks (Yu et al., 2018; Ferreira et al., 2019). The connection and aggregation of the network was decreased, and the compensatory ability of the network was reduced, which result in blocking of information transmission. Besides, some specific lesion areas in AD brain have been suggested by complex network analysis, and the roles of frontal lobe (FL), parietal lobe (PL) and basal ganglia (BG) in the development and progression of AD have only recently begun to receive attention. (Ferreira et al., 2019) found that the frontal cortex loss its modular connectivity with the subcortical gray matter structures and nodal global efficiency was decreased in the middle frontal cortex, which was associated with decreased motor function and cognitive function in AD patients. Srivishagan et al. (2020) applied the group measures to the structural connectomes of AD subjects based on the brain-lobes and demonstrated that the strength of the parietal lobes has been heavily affected in AD, and the impairment of the parietal lobe was reflected in the reduced ability to integrate sensations. Chen et al. (2022) found that AD patients predominantly decreased connectivity in the basal ganglia, demonstrating that the motor control and motor learning were declined.

**Figure 12:**
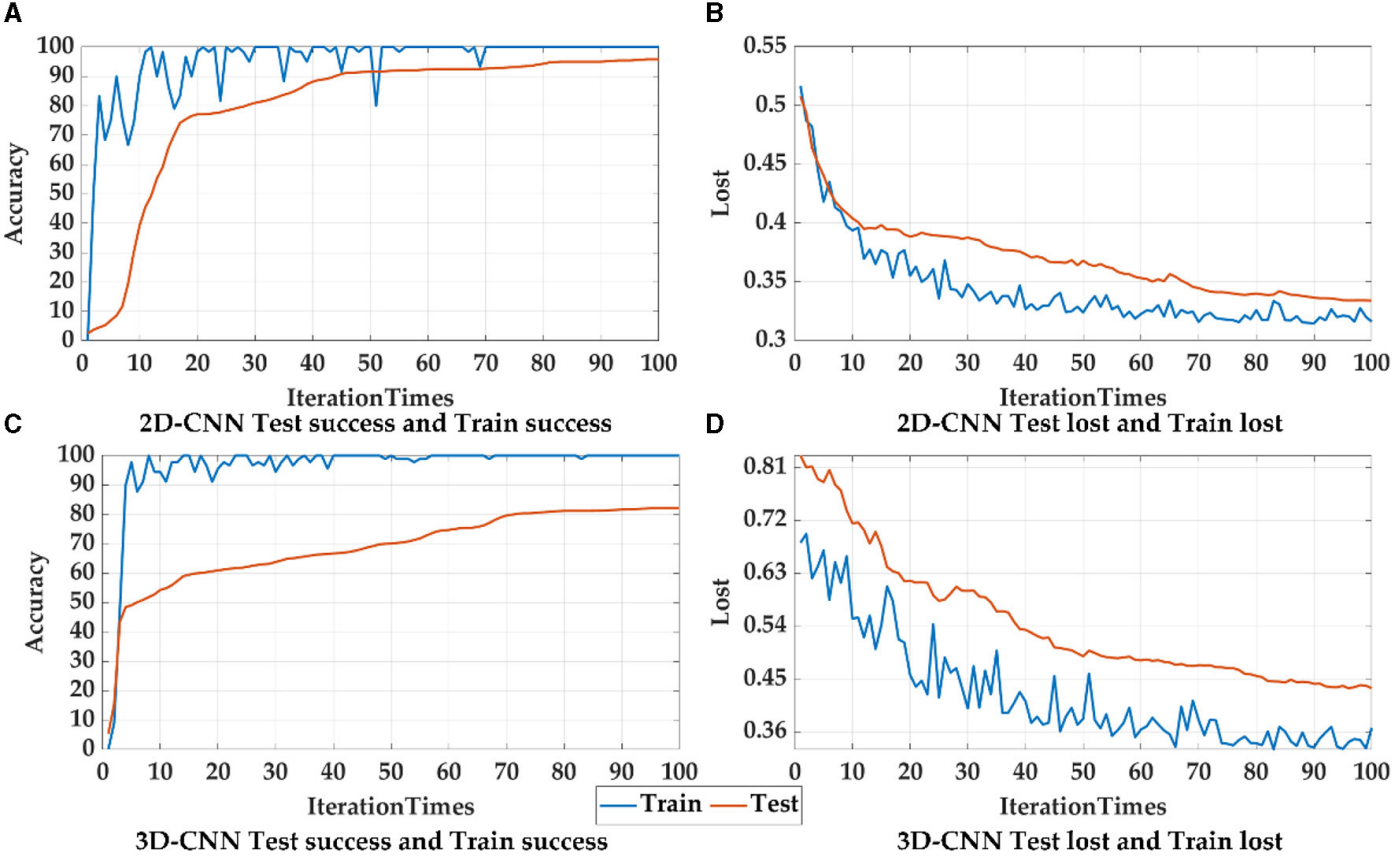
The accuracy and loss rates for training and testing convergence of 2D-CNN, and 3D-CNN, where blue curves represent the test set, and orange curves represent the training set.

Principally, CNN can identify and amplify these pathological changes and lesions on the network, due to the number of involved brain regions increases as the receptive field expands (Chen et al., 2019a). For lower layers of the CNN, a smaller receptive field may capture local patterns of the brain network, while a larger receptive field for higher layers may reflect global patterns. In CNN models, 2D-CNN and 3D-CNN are commonly used in the fMRI image recognition, so the above two CNNS were also used and compared in this paper, as shown in Figure 12, Table 8. Since 2D-CNN has only two dimensions, the convolutional kernel can move in both directions, which requires less convolutional computation and is more efficient compared to 3D-CNN. 2D-CNN can be used to amplify abnormalities in AD brain networks. In particular, the CNN structure was applied to PSI matrix (2D data) and fMRI data after pre-processing (3D data) for recognition, and the results of the accuracy and loss rate were showed in Figure 12, Table 8. For both types of CNNs, the test set shows the same trend as the training set with less fluctuations and smoother curves, and training accuracy is 100% after 100 iterations, which validates the effectiveness for both 2D-CNN and 3D-CNN. For the training set (Figures 12A, C), the 2D-CNN model could achieve 95.833% of test accuracy, which was higher than 3D-CNN; for the loss rate (Figures 12B, D), the loss rate of 2D-CNN was 33.396%, greatly lower than that of 3D-CNN. Clearly, 2D-CNN after constructing the brain network through PSI may achieve better recognition efficiency than 3D-CNN directly identifying pre-processed fMRI data.

**Table 8:** The recognition accuracy (ACC) and loss rate (LOSS) of 2D-CNN and 3D-CNN.

	**2D-CNN**		**3D-CNN**	
	**Train**	**Test**	**Train**	**Test**
ACC(%)	100 ± 0.00	95.83 ± 2.34	100 ± 0.00	82.11 ± 1.90
LOSS(%)	33.17 ± 3.36	33.39 ± 2.50	31.60 ± 3.36	43.21 ± 2.34

The analysis of the features shows eight deep features extracted from last convolutional layer in 2D-CNN had high correlations (*p* < 0.01) with seven topological features except AS (shown in Figure 12), thereby indicating that the global patterns described by the eight deep features were of certain importance in the AD identification problem and the CNN models excavated those patterns. However, it is still impossible to understand CNN from features alone, and the CNN model remains a black box. The underlying pathological relationship between these deep features and AD is still uncertain. As a result, further targeted clinical therapies cannot be pursued based on these findings. In contrast, with a precise methodological definition, topological features can reveal which lesions are present in the brain network of AD and can be helpful in assisting clinical diagnosis.

However, there were some limitations in this study. (1) The depth of the CNN model proposed in this paper and the parameters between the layers have optimization possibilities. (2) The courses of AD patients (LMCI, EMCI, et al.) was not taken into account, all classification results presented in this paper were two classifications for AD and HC. In the following studies, we will conduct more comprehensive analyses and use multivariate classification methods involving different AD processes. (3) It is important to acknowledge that overfitting was not extensively addressed in this study, which may limit the applicability of the proposed methods described in the paper.

In future work, we can enhance our research through various means, such as employing intelligent algorithms for parameter optimization of CNN, incorporating LSTM modules to optimize the structure of CNN, using Softmax activation function or multiple output nodes to perform multi-classification of different stages of Alzheimer's disease (AD) patients. At last, it would be valuable to explore additional strategies to combat overfitting, such as data augmentation techniques, dropout regularization, or ensemble methods. Considering the potential impact of overfitting on the reliability and generalizability of the results, it is important for future studies to prioritize addressing this issue and conducting thorough validation on independent datasets. By doing so, the validity and applicability of the proposed approach can be further substantiated.

## 6. Conclusion

In this paper, a new framework using PSI and 2D-CNN was applied on fMRI data in order to investigate the abnormalities of AD brain. Firstly, after pre-processing, the PSI analysis was applied to ROI signals for synchronization analysis thus mapped into a PSI network, and eight topological features were extracted. Compared with HC group, the brain network connectivity of AD group was sparser, connection strength was weaker, and the small-world property of the network was reduced. Specifically, the information transmission capacity and the rate of information transmission between network nodes of AD network was decreased. Secondly, the 2D-CNN was further applied to the PSI matrix to explore the local and global patterns of the brain network by extracting eight deep features from the 2D-CNN convolutional layers, which shows significant group differences by ANOVA analysis. Besides, the focus area could be exhibited from the feature map of 2D-CNN, such as frontal lobe (FL), parietal lobe (PL) and basal ganglia (BG). Then correlation analysis was implemented by Pearson analysis. As a result, topological features and deep features were correlated, verifying the possibility and effectiveness of the combination of the PSI and 2D-CNN methods. Finally, SVM was applied to classify AD fMRI by combing the PSI and 2D-CNN measures, which has the best performance (accuracy: 98.869%) compared to only using PSI or 2D-CNN.

The result reveals that the 2D-CNN model found some deep features that showed global patterns and texture patterns representing the abnormal connectivity in the brain network of AD patients and other patterns that could not described by the topological features. This paper demonstrates that 2D-CNN has the amazing learning ability and could identify potential biases in AD patients by extracting deep features of convolutional layers. Those features may provide new insights into the underlying pathogenesis of AD.

## Data availability statement

Publicly available datasets were analyzed in this study. This data can be found here: Alzheimer's Disease Neuroimaging Initiative: ADNI https://adni.loni.usc.edu/.

## Ethics statement

Written informed consent was obtained from the individual(s) for the publication of any potentially identifiable images or data included in this article.

## Author contributions

RW, QH, and HW designed the study and analyzed the results. RW, QH, and LS provided the code support of algorithm. QH carried out the experiments. RW and QH wrote the original draft. RW, QH, CH, and YC polished the sentences of the article. RW, QH, HW, and YC modified the format of figures and tables. QH and HW finalize format of the manuscript. RW, CH, and YC provided the funding. All authors contributed to the article and approved the submitted version.
